# Retraction: Long non-coding RNA TUSC7, a target of miR-23b, plays tumor-suppressing roles in human gliomas

**DOI:** 10.3389/fncel.2026.1834443

**Published:** 2026-03-25

**Authors:** Frontiers Editorial Office

The Journal retracts the 2016 article cited above.

Following publication, concerns were raised on PubPeer regarding the integrity of the images in the published figures. Specifically, there are multiple overlapping sections across Figures 3D, 5B, and 6B. The authors failed to provide a satisfactory explanation during the investigation, which was conducted in accordance with Frontiers' policies.

In addition, during the course of this investigation, the authors identified that the U87 cell line used in this study was contaminated with HeLa cells.

As a result, the data and conclusions of the article have been deemed unreliable and the article has been retracted.

This retraction was approved by the Chief Executive Editor of Frontiers. The authors agree to this retraction.

